# Tocopherols in Sunflower Seedlings under Light and Dark Conditions

**DOI:** 10.1155/2015/146782

**Published:** 2015-08-12

**Authors:** Lidia del Moral, Begoña Pérez-Vich, Leonardo Velasco

**Affiliations:** Instituto de Agricultura Sostenible (IAS-CSIC), Alameda del Obispo, s/n, 14004 Córdoba, Spain

## Abstract

The objective of this study was to evaluate the dynamics of tocopherols in cotyledons and radicles from sunflower seeds with high and low total tocopherol content, mainly in the *α*-tocopherol form, and from seeds with increased proportions of *β*-, *γ*-, and *δ*-tocopherol, both under dark and light conditions. Tocopherol content was measured every 24 h from 1 to 12 days after sowing. In all cases, the content of individual tocopherol forms in the cotyledons and radicles was reduced along the sampling period, which was more pronounced under light conditions. The presence of light had a slightly greater effect on *α*- and *γ*-tocopherol than on *β*- and *δ*-tocopherol. A marked light effect was also observed on total tocopherol content, with light promoting the reduction of tocopherol content in cotyledons and radicles. The study revealed only slight differences in the patterns of tocopherol losses in lines with different tocopherol profiles, both under dark and light conditions, which suggested that the partial replacement of *α*-tocopherol by other tocopherol forms had no great impact on the protection against oxidative damage in seedlings.

## 1. Introduction

Tocopherols are lipophilic antioxidants synthesized by photosynthetic organisms such as plants and some algae and cyanobacteria. Their chemical structure is formed by a chromanol ring and a saturated phytyl-derived side chain. Depending on the number and positions of methyl groups in the chromanol ring, they are named as *α*-, *β*-, *γ*-, and *δ*-tocopherol [1]. In general, *α*-tocopherol is the predominant tocopherol form in photosynthetic tissues, while *γ*- and *δ*-tocopherol are predominant in seeds, fruits, and storage organs of dicots [2, 3]. Seeds of monocots contain varying proportions of tocopherols and tocotrienols, the latter being similar to tocopherols except for having three unsaturated bonds in the side chain [2]. Tocopherols protect photosynthetic membranes from photosynthesis-derived reactive oxygen species, limiting subsequently the extent of lipid peroxidation [4]. Tocopherols are also involved in plant cell protection from oxidative damage under abiotic stress conditions [5].

Sunflower seeds contain predominantly *α*-tocopherol in the seeds instead of *γ*- and *δ*-tocopherol, which are the most common tocopherol homologues in seeds [3]. Several sunflower mutants in which seed *α*-tocopherol content was partially to nearly completely replaced by other tocopherol homologues have been identified [6–8]. Also, sunflower lines with increased and reduced total content of *α*-tocopherol in seeds have been reported [9]. Modifications of seed tocopherols were also reflected at other plant levels. In particular, increased levels of *β*-, *γ*-, and *δ*-tocopherol in the seeds were paralleled by increased levels of these tocopherol forms in roots, leaves, and pollen grains [10]. A line with reduced total content of *α*-tocopherol in seeds also showed a reduced content in roots and pollen grains [10].

There are no studies on the evolution of seed tocopherols in sunflower shortly after seed germination and how light conditions may influence this process. This is important because of the role of seed tocopherols in protecting plant tissues from lipid oxidation during germination and early seedling development [11]. The large variability for seed tocopherol content and profile available in sunflower provides an exceptional material for studying how tocopherols change shortly after germination and how this process may be affected by the presence or absence of light. Accordingly, the objective of this research was to study the contents of individual tocopherols in the radicles and cotyledons of ten sunflower lines with contrasting seed tocopherols levels between 1 and 12 days after sowing (DAS) under dark and light conditions.

## 2. Materials and Methods

### 2.1. Plant Materials

Ten lines with contrasting seed tocopherols levels were used. Lines IAST-306 and IAST-413, with high seed tocopherol content, and line IAST-522, with low seed tocopherol content, in the three cases in the *α*-tocopherol form, were developed by selection from germplasm accessions [9]. Lines T2100 and T589, with increased levels of *γ*-tocopherol and *β*-tocopherol, respectively, were isolated from germplasm accessions [7]. Line IAST-1, with high gamma-tocopherol content, was obtained by chemical mutagenesis [8]. Lines IAST-4, with increased levels of both *γ*- and *δ*-tocopherol, and IAST-5, with increased levels of *β*-tocopherol, were isolated from crosses between lines IAST-1 and T589 [8]. Line TS-1, with increased levels of both gamma- and delta-tocopherol content, was also derived from crosses between IAST-1 and T589 (L. Velasco, unpublished). Line HA89 is a line with tocopherol content and profile typical of sunflower cultivars [9].  Table 1 shows seed tocopherols contents of the ten lines included in the study.

### 2.2. Sampling

Around 250 seeds of each line were placed on moistened filter paper in Petri dishes that were maintained in a growth chamber at 25/20°C (day/night) with a 16-hour photoperiod and photon flux density of 300 *µ*mol m^−2^ s^−1^. The Petri dishes were wrapped with two layers of aluminum foil to prevent light intrusion. After 24 hours, half of the Petri dishes were unwrapped for light treatment. Hulls were removed as seeds were germinating.

Seeds were sampled at 1, 2, 3, 4, 5, 6, 8, 10, and 12 days after sowing (DAS), both under light and dark conditions. Three replicates per line and light treatment were collected at each sampling time, each consisting of five seeds. Seeds of each replication were immediately divided into cotyledons and radicles, which were stored separately in Eppendorf tubes at −80°C. After lyophilisation, the tissue was ground to a fine powder with a glass rod and analysed for tocopherol content as described below.

### 2.3. Tocopherol Analyses

Tocopherol extraction and analysis by high-performance liquid chromatography (HPLC) were performed following a previously reported procedure [12], using a fluorescence detector (Waters 474) at 295 nm excitation and 330 nm emission and isooctane/tert-butylmethylether (94 : 6) as eluent at an isocratic flow rate of 0.8 mL/min. Chromatographic separation of the tocopherols was performed on a LiChrospher 100 diol column (250 mm × 2 mm I.D.) with 5 *μ*m spherical particles, connected to a silica guard column (LiChrospher Si 60, 5 mm × 4 mm I.D.). Quantitative determination of tocopherols was performed using external calibration curves obtained for each of the tocopherol homologues, alpha-, beta-, gamma-, and delta-tocopherol (Calbiochem Tocopherol Set, Cat. number 613424, Merck KGaA, Darmstadt, Germany). Tocopherol content for each of the tocopherol forms was expressed as mg kg^−1^ seed kernel. Total tocopherol content was calculated as the sum of the four tocopherol forms.

### 2.4. Statistical Analysis

Data were analysed by the General Linear Model procedure of IBM SPSS Statistics version 20 (IBM Corp., Armonk, NY, USA) using a factorial model with four main fixed factors (genotype, light treatment, tissue, and DAS) and their corresponding interactions. The areas under the tocopherol curves were computed using Origin Pro 9.1.0 (OriginLab Corporation, Northampton, MA, USA). Since light treatment started at 1 DAS, the analysis of variance focused on data from 2 to 12 DAS. Comparison of the areas obtained under dark and light conditions was done by paired *t*-tests using Origin Pro 9.1.0.

## 3. Results

The four main factors, genotype, tissue, light, and DAS, as well as most of their interactions influenced significantly the contents of individual tocopherols and the total tocopherol content (Table 2). Most of the variation for the content of individual tocopherols, estimated as the percentage of sums of squares, corresponded to the genotype followed by the tissue. Light had greater effect on *α*- and *γ*-tocopherol than on *β*- and *δ*-tocopherol contents. For total tocopherol content, tissue had the main effect followed by genotype, light, and DAS. In all cases, the main interaction was between genotype and tissue (Table 2).

**Figure 1:**
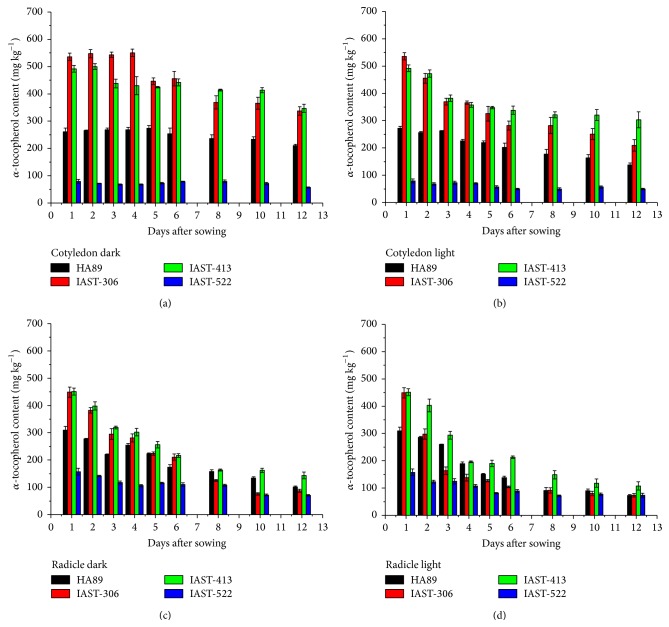
*α*-tocopherol content (mg kg^−1^ dry tissue) from 1 to 12 days after sowing in cotyledons and radicles of sunflower lines HA89, IAST-306, IAST-413, and IAST-522, with contrasting levels of seed tocopherol content predominantly in the *α*-tocopherol form, from seedlings grown under dark and light conditions. Data are presented as mean ± standard error of three replications.

**Figure 2:**
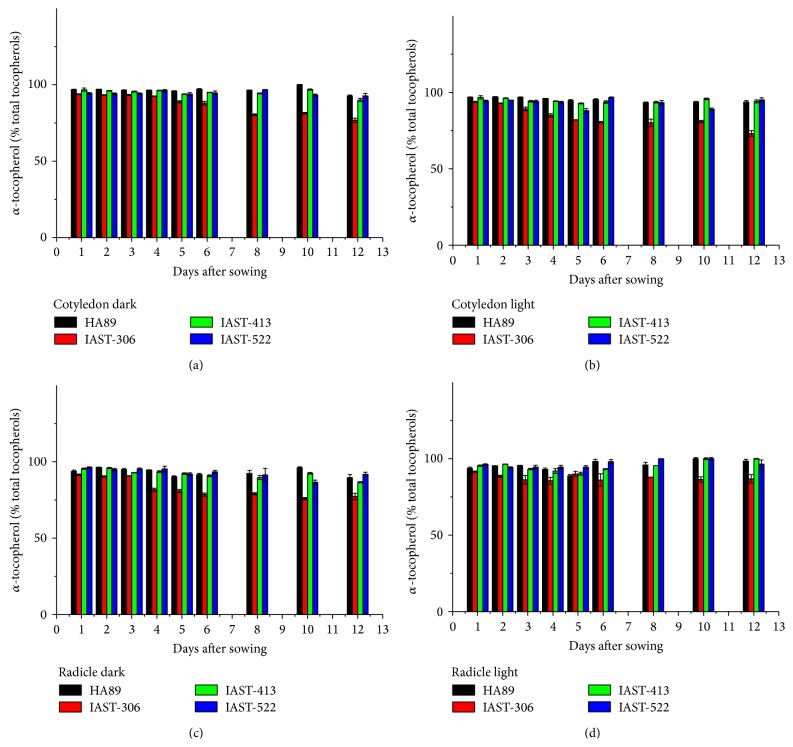
Proportion of *α*-tocopherol (% total tocopherols) from 1 to 12 days after sowing in cotyledons and radicles of sunflower lines HA89, IAST-306, IAST-413, and IAST-522, with contrasting levels of seed tocopherol content predominantly in the *α*-tocopherol form, from seedlings grown under dark and light conditions. Data are presented as mean ± standard error of three replications.

In the four lines with conventional tocopherol profile, *α*-tocopherol content decreased along the sampling period, particularly in the radicles (Figure 1). The reduction of tocopherol concentration was more pronounced under light conditions. The effect was more marked in the two lines with high tocopherol content, IAST-306 and IAST-413. Thus, for example, *α*-tocopherol content at 12 DAS in the cotyledons of line IAST-306 was 337 mg kg^−1^ under dark and 210 mg kg^−1^ under light conditions, compared to 536 mg kg^−1^ at 1 DAS. In the radicles, *α*-tocopherol content dropped from 449 mg kg^−1^ at 1 DAS to 87 mg kg^−1^ at 12 DAS under dark and 73 mg kg^−1^ under light conditions. The tocopherol profile was dominated by *α*-tocopherol in the four lines throughout the sampling period (Figure 2). Amongst the four lines, only line IAST-306 showed a marked reduction in the proportion of *α*-tocopherol in the last sampling dates, which was mainly replaced by *γ*-tocopherol.

**Figure 3:**
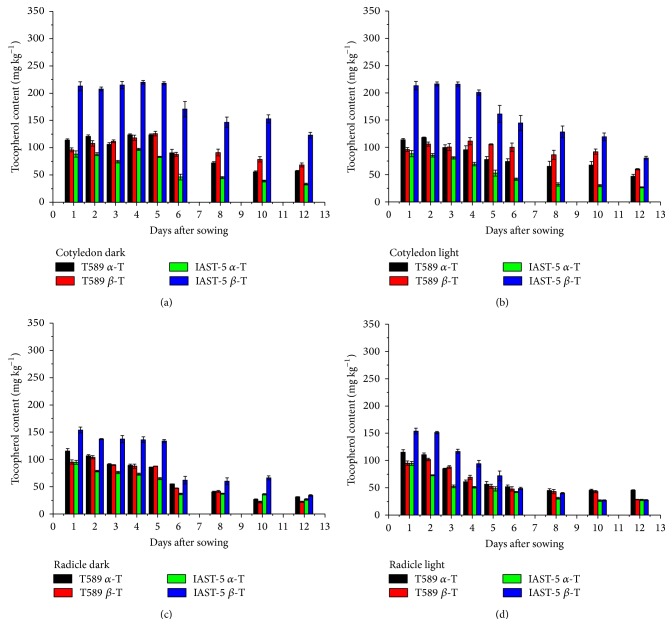
*α*- and *β*-tocopherol contents (mg kg^−1^ dry tissue) from 1 to 12 days after sowing in cotyledons and radicles of sunflower lines T589 and IAST-5, with different increased levels of *β*-tocopherol, from seedlings grown under dark and light conditions. Data are presented as mean ± standard error of three replications.

**Figure 4:**
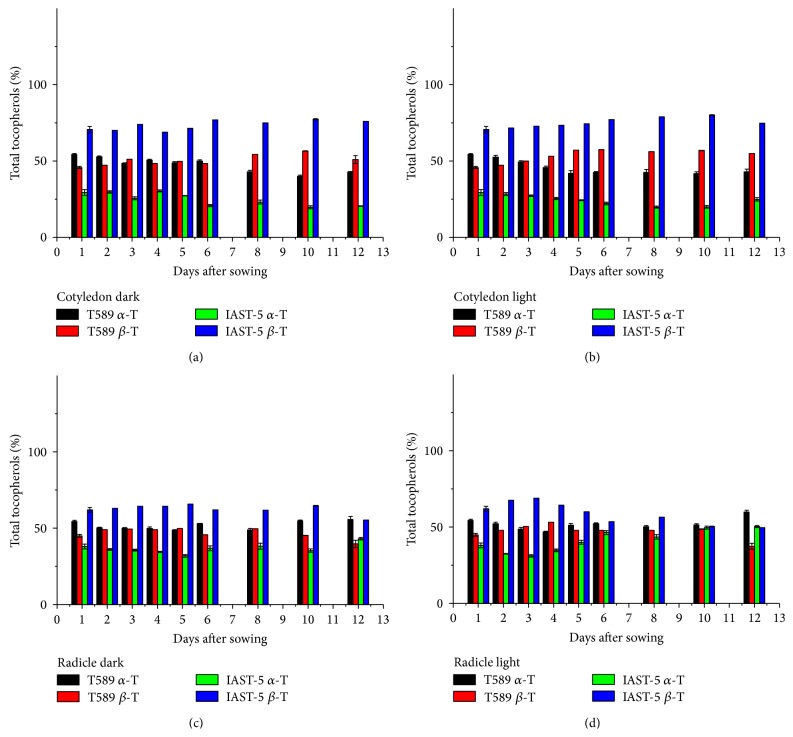
Proportion of *α*- and *β*-tocopherol (% total tocopherols) from 1 to 12 days after sowing in cotyledons and radicles of sunflower lines T589 and IAST-5, with different increased levels of *β*-tocopherol, from seedlings grown under dark and light conditions. Data are presented as mean ± standard error of three replications.

In the two lines with increased levels of *β*-tocopherol, the content of both *α*-tocopherol and *β*-tocopherol decreased along the sampling period (Figure 3). For instance, *β*-tocopherol dropped from 213 mg kg^−1^ at 1 DAS to 123 mg kg^−1^ (dark) and 80 mg kg^−1^ (light) at 12 DAS in the cotyledons and from 154 mg kg^−1^ at 1 DAS to 34 mg kg^−1^ (dark) and 27 mg kg^−1^ (light) at 12 DAS in the radicles of line IAST-5. In the cotyledons of both lines, the proportion of *α*-tocopherol decreased and that of *β*-tocopherol increased along the sampling period, although the changes were of small magnitude (Figure 4). In line T589, changes in the relative proportions of *α*-tocopherol and *β*-tocopherol in the cotyledons were observed earlier under light conditions.

**Figure 5:**
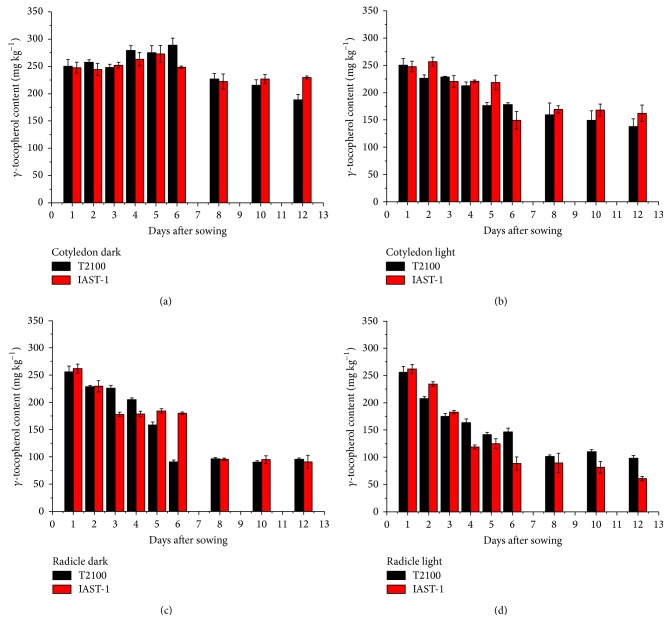
*γ*-tocopherol content (mg kg^−1^ dry tissue) from 1 to 12 days after sowing in cotyledons and radicles of sunflower lines T2100 and IAST-1, with seed tocopherol content predominantly in the *γ*-tocopherol form, from seedlings grown under dark and light conditions. Data are presented as mean ± standard error of three replications.

**Figure 6:**
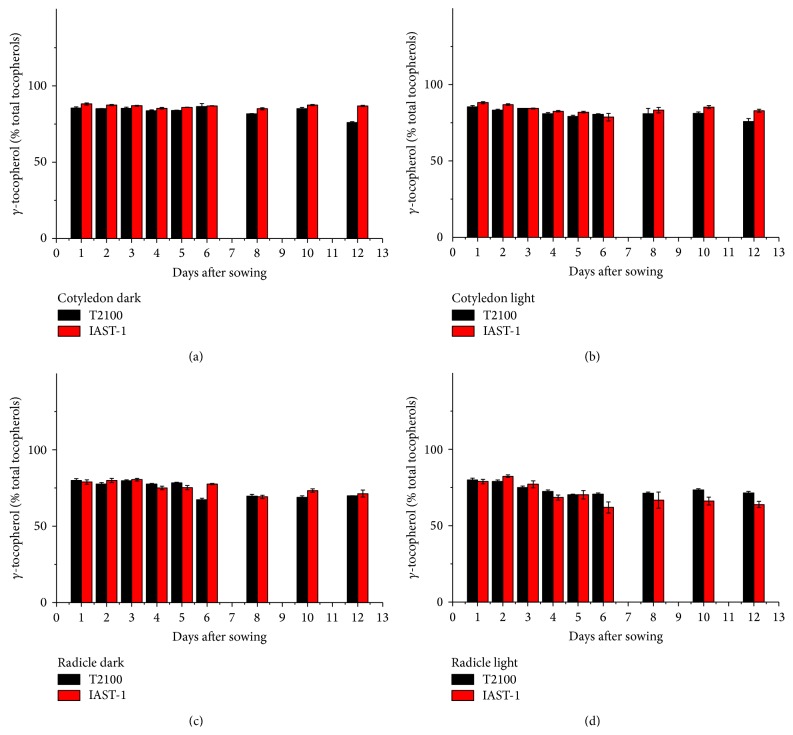
Proportion of *γ*-tocopherol (% total tocopherols) from 1 to 12 days after sowing in cotyledons and radicles of sunflower lines T2100 and IAST-1, with seed tocopherol content predominantly in the *γ*-tocopherol form, from seedlings grown under dark and light conditions. Data are presented as mean ± standard error of three replications.

**Figure 7:**
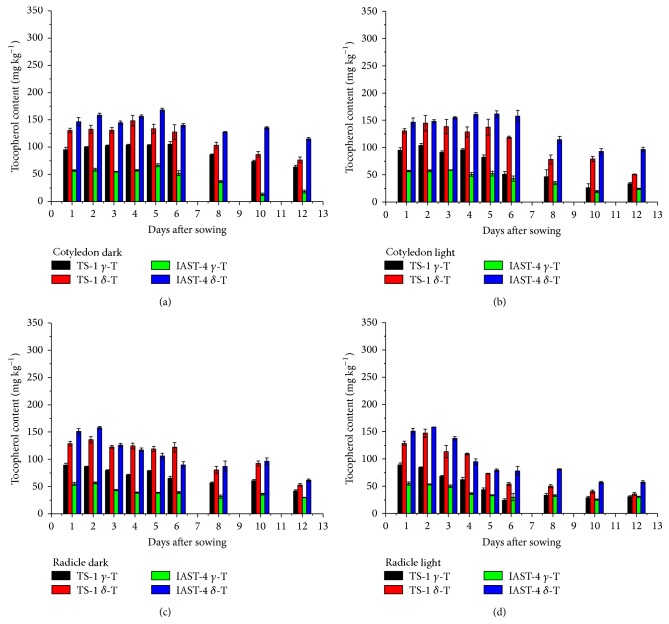
*γ*- and *δ*-tocopherol contents (mg kg^−1^ dry tissue) from 1 to 12 days after sowing in cotyledons and radicles of sunflower lines TS-1 and IAST-4, with different increased levels of *γ*- and *δ*-tocopherol, from seedlings grown under dark and light conditions. Data are presented as mean ± standard error of three replications.

**Figure 8:**
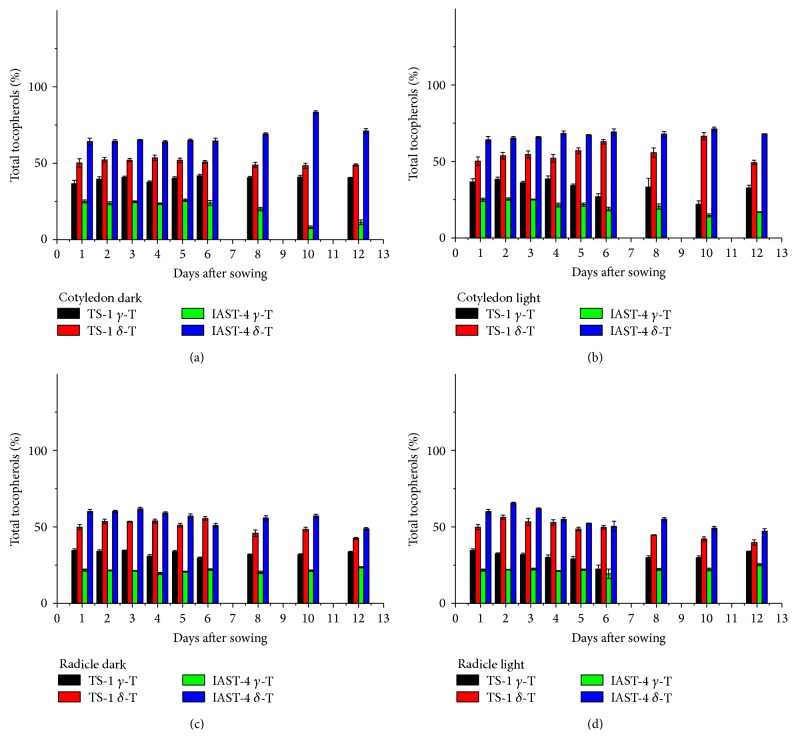
Proportions of *γ*- and *δ*-tocopherol (% total tocopherols) from 1 to 12 days after sowing in cotyledons and radicles of sunflower lines TS-1 and IAST-4, with different increased levels of *γ*- and *δ*-tocopherol, from seedlings grown under dark and light conditions. Data are presented as mean ± standard error of three replications.

In the two lines with high *γ*-tocopherol content, IAST-1 and T2100, the concentration of this tocopherol in the cotyledons increased slightly till 5 or 6 DAS under dark conditions and then decreased. This was not observed under light conditions (Figure 5). Considering the whole sampling period, *γ*-tocopherol in the cotyledons of line T2100 decreased from 250 mg kg^−1^ at 1 DAS to 189 mg kg^−1^ (dark) and 138 mg kg^−1^ (light) at 12 DAS, whereas it decreased from 248 mg kg^−1^ at 1 DAS to 230 mg kg^−1^ (dark) and 162 mg kg^−1^ (light) at 12 DAS in line IAST-1. A marked reduction of *γ*-tocopherol concentration was also observed in the radicles of both lines (Figure 5). In both tissues, reduction of *γ*-tocopherol levels was not paralleled by changes in other tocopherol forms such as *α*- or *δ*-tocopherol, with the high proportion of *γ*-tocopherol remaining practically unchanged (Figure 6). The two lines with increased levels of both *γ*- and *δ*-tocopherol showed also a significant reduction of both tocopherol forms in the cotyledons and radicles during the sampling period (Figure 7). In the cotyledons, such a reduction only started after 5 or 6 DAS under dark conditions. Similarly to the other lines evaluated in this study, changes in the tocopherol profile during the sampling period were of small magnitude and somewhat inconsistent. For instance, the relative proportion of *γ*-tocopherol increased in the cotyledons of line TS-1 but decreased in those of IAST-4 under dark conditions (Figure 8).

**Figure 9:**
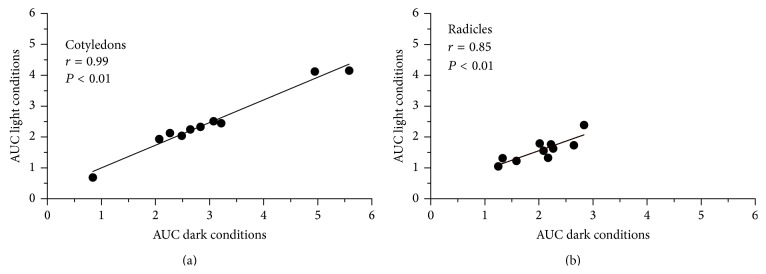
Scatter plots of the area under the curve (AUC, arbitrary units ×10^−3^) of total tocopherol content from 1 to 12 days after sowing in cotyledons and radicles of ten sunflower lines under dark and light conditions.

Light influence on tocopherol content of sunflower seedlings was further studied using total tocopherol content instead of the contents of individual tocopherols, due to the impracticability of inferring statistical significance in cases such as *β*- and *δ*-tocopherol, for which only two lines with increased levels were available. The areas under the tocopherol curves were computed and averaged for each type of tissue and light conditions using the ten lines included in the study. Cotyledons under dark conditions had an average area under the curve (AUC) of 2994 ± 1374 (arbitrary AUC units, given as mean ± standard deviation), compared to 2462 ± 1021 under light conditions. Comparison of means revealed that the difference was significant (*t* = 4.21, *P* < 0.01). Similarly, radicles under dark conditions had an average AUC of 2039 ± 521, compared to 1579 ± 380 under light conditions (*t* = 5.13, *P* < 0.01). Despite such significant differences, the AUC values under dark and light conditions were highly correlated both for cotyledons (*r* = 0.99) and for radicles (*r* = 0.85) (Figure 9).

## 4. Discussion

This study analyzed for the first time the dynamics of tocopherols during the initial stages of seedling development in a set of sunflower lines with contrasting levels of individual tocopherols as well as total tocopherol content. Our results showed that tocopherol content in sunflower seedlings decreased during the initial stages of seedling development, both in the cotyledons and in the radicles. Such a reduction was more pronounced under light conditions. Despite the reduction in total tocopherol content, changes in the tocopherol profile were in general of small magnitude. There are no previous studies on the dynamics of tocopherol content in sunflower seedlings shortly after germination. Previous reports in other species have shown contrasting results, with the pattern of tocopherol mobilization during germination being dependent on the plant species. In Brassicaceae, studies carried out in rapeseed (*Brassica napus* L.), Ethiopian mustard, white mustard (*Sinapis alba* L.), or radish (*Raphanus sativus* L.) revealed a general trend towards an increase of *α*-tocopherol during the initial steps of seedling development [13–15]. However, no consistent changes in *γ*-tocopherol have been reported. Thus, whereas in some cases *γ*-tocopherol was found to increase during 3-4 DAS to decrease afterwards, in other cases *γ*-tocopherol content decreased gradually from a maximum content found in dry seeds [13–15]. In legumes, analysis of changes in tocopherols during germination in mung bean (*Vigna radiata* (L.) R. Wilczek), soybean (*Glycine max* (L.) Merr.), and lupin (*Lupinus albus* L.) showed in general an increase in *γ*-tocopherol content (the major tocopherol form) during the first days after sowing (from 0 to 3–5 DAS, depending on the species and even the cultivar), followed by a significant decrease until the end of the sampling period (from 4 to 7 DAS) [16–18]. Most of the studies also showed an increase in *α*- and *δ*-tocopherol during the first days after sowing (from 0 to 3–5 DAS), excepting a study in soybean in which *α*-tocopherol levels were low and remained constant during the germination process [18].

There are contradictory results in the literature on the influence of light on tocopherol content in developing seedlings. For example, Zielinski and Kozlowska [13] found that replacement of *γ*- by *α*-tocopherol occurred more rapidly under light conditions in the seedlings of several cruciferous crops. Conversely, Shi et al. in soybean [18] and García-Navarro et al. in Ethiopian mustard [15] found no detectable light effect on the dynamics of tocopherols in the seedlings. In the present research, a marked light effect was observed, with light conditions promoting the reduction of tocopherol content in sunflower seedlings, both in the cotyledons and in the radicles. Light effect was consistent across ten different lines with contrasting tocopherol content and/or profile, which points to similar responses of the different tocopherol homologues under light conditions in sunflower seedlings. Photosynthesis in the chloroplasts produces reactive oxygen species as a mechanism to dissipate excess of energy. In this process, tocopherols act protecting membrane lipids from peroxidation caused by the reactive oxygen species by controlling nonenzymatic oxidation of polyunsaturated fatty acids [1]. Chemical scavenging of reactive oxygen species by *α*-tocopherol leads to a net *α*-tocopherol loss, due to the formation of *α*-tocopherol oxidation products [4]. This can explain the results obtained in the present research in the lines containing *α*-tocopherol. Studies on the* in vivo* protective role of other tocopherol forms are scarce, since *α*-tocopherol is ubiquitously predominant in photosynthetic tissues [2]. In a few mutant [19] and natural plants [20] in which *γ*-tocopherol is the predominant form in photosynthetic tissues, no detrimental effects on oxidative stress protection have been observed associated with the presence of *γ*- instead of *α*-tocopherol.

## 5. Conclusions

Our results revealed similar patterns of tocopherol losses in lines with different tocopherol profiles, both under dark and light conditions, with very slight differences in the *γ*- and *δ*-tocopherol lines. The high correlation coefficients between AUC values under light and dark conditions in a set of lines with contrasting tocopherol profiles suggested that the* in vivo* protective action of tocopherols against lipid oxidation at the seedling level was not dependent on the tocopherol profile, though this should be confirmed through more specific studies.

## Figures and Tables

**Table 1:** *α*-, *β*-, *γ*-, *δ*-, and total tocopherol (T) content (mg kg^−1^ dry seed kernel given as mean ± SD) in nine sunflower lines with modified seed tocopherol levels and the control line HA89^1^.

Line	Alpha-T	Beta-T	Gamma-T	Delta-T	Total T
	mg kg^−1^ seed				
HA89	263.3 ± 46.1^d^	2.6 ± 2.2^a^	ND^2^	ND	266.0 ± 45.9^cd^
IAST-522	148.6 ± 13.5^c^	4.9 ± 1.7^a^	ND	ND	153.5 ± 13.1^a^
IAST-306	472.8 ± 34.6^e^	40.3 ± 16.8^b^	ND	ND	513.3 ± 38.0^e^
IAST-413	472.6 ± 44.9^e^	10.7 ± 4.8^a^	6.8 ± 9.7^a^	ND	490.1 ± 51.1^e^
T589	77.9 ± 10.2^b^	128.5 ± 39.9^c^	ND	ND	206.4 ± 48.4^ab^
IAST-5	52.9 ± 10.7^b^	222.8 ± 47.8^d^	ND	ND	275.6 ± 51.9^cd^
T2100	0.3 ± 1.3^a^	ND	300.9 ± 39.3^c^	3.3 ± 2.3^a^	304.6 ± 39.8^d^
IAST-1	1.4 ± 0.7^a^	ND	269.2 ± 61.9^c^	3.3 ± 2.3^a^	273.9 ± 63.5^cd^
TS-1	5.0 ± 4.8^a^	2.2 ± 3.9^a^	86.8 ± 13.0^b^	144.0 ± 57.6^b^	238.0 ± 58.0^bc^
IAST-4	2.6 ± 1.0^a^	2.8 ± 3.4^a^	54.9 ± 10.8^b^	171.4 ± 28.4^c^	231.6 ± 34.8^bc^

^1^Means with different letters within each tocopherol homologue differ significantly (*P* < 0.05) based on one-way ANOVA and Tukey's range test.

^2^Not detectable.

**Table 2:** Analysis of variance (percentage of total mean squares) for *α*-, *β*-, *γ*-, *δ*-, and total tocopherol (T) content (mg kg^−1^ dry tissue) in cotyledons and roots of seedlings from ten sunflower lines grown under light and dark conditions from 2 to 12 days after sowing (DAS).

Source of variation	*α*-T	*β*-T	*γ*-T	*δ*-T	Total T
Genotype (G)	61.89^*∗∗*^	70.00^*∗∗*^	74.60^*∗∗*^	87.74^*∗∗*^	18.34^*∗∗*^
Tissue (T)	15.61^*∗∗*^	16.59^*∗∗*^	14.33^*∗∗*^	6.48^*∗∗*^	50.39^*∗∗*^
Light (L)	6.18^*∗∗*^	1.13^*∗∗*^	4.05^*∗∗*^	0.63^*∗∗*^	12.84^*∗∗*^
DAS (D)	4.54^*∗∗*^	3.61^*∗∗*^	1.10^*∗∗*^	1.74^*∗∗*^	10.12^*∗∗*^
G × T	8.60^*∗∗*^	6.72^*∗∗*^	3.19^*∗∗*^	1.67^*∗∗*^	4.99^*∗∗*^
G × L	1.30^*∗∗*^	0.42^*∗∗*^	0.65^*∗∗*^	0.33^*∗∗*^	0.73^*∗∗*^
G × D	0.65^*∗∗*^	1.02^*∗∗*^	0.63^*∗∗*^	0.72^*∗∗*^	0.23^*∗∗*^
T × L	0.42^*∗∗*^	0.01^ns^	0.23^*∗∗*^	0.03^ns^	0.54^*∗∗*^
T × D	0.27^*∗∗*^	0.15^*∗∗*^	0.34^*∗∗*^	0.26^*∗∗*^	0.84^*∗∗*^
L × D	0.22^*∗∗*^	0.12^*∗∗*^	0.21^*∗∗*^	0.07^*∗∗*^	0.53^*∗∗*^
G × T × L	0.11^*∗∗*^	0.02^ns^	0.30^*∗∗*^	0.04^*∗*^	0.10^*∗∗*^
G × T × D	0.08^*∗∗*^	0.05^*∗∗*^	0.13^*∗∗*^	0.12^*∗∗*^	0.07^*∗∗*^
G × L × D	0.06^*∗∗*^	0.09^*∗∗*^	0.12^*∗∗*^	0.09^*∗∗*^	0.07^*∗∗*^
T × L × D	0.06^*∗∗*^	0.03^ns^	0.06^*∗∗*^	0.03^ns^	0.15^*∗∗*^
G × T × L × D	0.02^ns^	0.03^*∗*^	0.03^*∗∗*^	0.02^ns^	0.03^ns^
Error	0.02	0.02	0.02	0.02	0.03

^*∗∗*,*∗*^Significant at the 1% and 5% probability levels, respectively.

ns: not significant.

## Abbreviations

DAS: Days after sowing
AUC: Area under the curve.
